# Prognostic value of nicotinamide *N*-methyltransferase in human cancers: Evidence from a meta-analysis and database validation

**DOI:** 10.1515/med-2022-0413

**Published:** 2022-02-14

**Authors:** Ling Dang, Yingdong Wang

**Affiliations:** Department of Digestive Internal Medicine, People’s Hospital of Tongchuan, Tongchuan, Shaanxi, 727000, China; Department of Abdominal Oncology, Shaanxi Provincial Cancer Hospital, Xi’an, Shaanxi, 710061, China

**Keywords:** nicotinamide *N*-methyltransferase, cancer, prognosis, meta-analysis, TCGA

## Abstract

**Background:**

Previous studies indicated that dysregulated expression of nicotinamide *N*-methyltransferase (NNMT) contributed to the tumor progression and predicted poor prognosis in various cancers. However, there was no exact conclusion on account of the contradictory results across studies.

**Methods:**

The relevant studies up to December 7, 2020 were searched in PubMed, Web of Science, and Embase. The association between NNMT expression and prognostic outcomes was explored, including overall survival (OS), disease-free survival (DFS), and clinicopathological features. The bioinformatics database was used to validate the findings.

**Results:**

Thirteen retrospective studies containing 2,591 patients with cancers were included in this analysis. High NNMT expression was significantly associated with shorter OS (hazard ratio [HR] = 2.01, 95% confidence interval [CI] = 1.42–2.86, and *P* < 0.01) and DFS (HR = 1.59, 95% CI = 1.23–2.05, and *P* < 0.01) compared to low NNMT expression in cancers. Compared to patients with low NNMT expression, patients with high NNMT expression tended to have worse tumor differentiation (*P* = 0.03), earlier lymph node metastasis (*P* = 0.01), earlier distant metastasis (*P* = 0.02), and more advanced clinical stage (*P* < 0.01).

**Conclusion:**

High NNMT expression is an unfavorable factor of various cancers. NNMT is a promising indicator to predict the prognosis of various cancers and can serve as a potential therapeutic target in various cancers.

## 1 Introduction

Cancer has become one of the major public health concerns globally and the leading cause of death [1]. It is estimated that there were 1,688,780 new cases of cancer and approximately 600,920 deaths resulting from cancer in 2017 in the United States [2]. Despite the great improvement in diagnosis and treatment, many patients suffered from a poor prognosis, especially those at advanced clinical stage [1]. To solve this dilemma, increasing number of researchers are devoted to establishing new potential biomarkers to improve the clinical decision-making and prolong the survival time of cancer patients [3,4].

Nicotinamide *N*-methyltransferase (NNMT) is a cytosolic enzyme that catalyzes the pyridine compounds and *N*-methylation of nicotinamide [5]. Recently, accumulating evidence showed that abnormal expression of NNMT might play an important role in the tumorigenesis, invasion, and metastasis [6,7,8]. Previous studies have showed that NNMT expression had the potential capacity to predict the prognosis of several cancers, such as endometrial cancer [9], pancreatic cancer [10], and gastric cancer [11]. However, definite conclusion about the prognostic value of NNMT expression in human cancers has not been determined on account of contradictory results among existing evidence [9,10,11,12,13,14,15,16,17,18,19,20,21]. A study by Akar et al. showed that the overexpression of NNMT was associated with the aberrant p53 expression, pAkt, and poor survival (*P* = 0.03) [9]. Similarly, Chen et al. also found that overexpression of NNMT might predict the shorter survival time (*P* = 0.03) and be associated with worse clinicopathological features (e.g., distant metastasis and clinical stage) in gastric cancer [11]. However, a different voice from the study of Bi et al. showed that there was no significant relationship between NNMT expression and survival time in pancreatic cancer [10]. Therefore, there is a dispute on the prognostic value of NNMT expression in cancers based on existing evidence [9,10,11,12,13,14,15,16,17,18,19,20,21]. To settle this dispute, we performed this meta-analysis by integrating the existing evidence and validated our findings using the bioinformatics database.

## 2 Materials and methods

Ethical approval and informed consent were unnecessary because no data of individuals were used in this research.

### 2.1 Literature search

This study was performed according to Preferred Reporting Items for Systematic Reviews and Meta-Analyses [22]. PubMed, Web of Science, and Embase were comprehensively researched for relevant studies on December 26, 2020. The following search strategy was used: (“nicotinamide *N*-methyltransferase” OR “NNMT”) AND (“cancer” OR “tumor” OR “carcinoma” OR “neoplasm”) AND (“survival” OR “prognosis” OR “predict”). Potential related studies were also searched in the references of the retrieved articles.

### 2.2 Study inclusion and exclusion criteria

Inclusion criteria for selecting the studies were as follows: (1) patients were diagnosed with any type of cancer; (2) studies explored the association of NNMT expression with clinicopathological parameters (CP) and survival outcomes of cancer patients, such as overall survival (OS), cancer-specific survival (CSS), disease-free survival (DFS), and recurrence-free survival (RFS); (3) studies had the retrospective or prospective design; and (4) studies were published in English. Exclusion criteria were as follows: (1) reviews, case reports, or cell studies; (2) studies without sufficient data; (3) duplication or studies containing duplicated patients; and (4) data of patients were from common bioinformatics database. The study selection was performed by the abovementioned criteria by two authors independently, and disagreement were solved by discussion.

### 2.3 Data extraction and quality assessment

We extracted the following information of each study using a prepared template: (1) information of the first author, publication year, country, total number of patients, number of patients with high or low NNMT expression, definition of high NNMT expression, detection methods, source of sample, outcomes, analysis model of OS, and adjusted factors in the multivariate analysis; (2) CP including age, gender, tumor size, tumor differentiation, T stage, lymph node metastasis, distant metastasis, and clinical stage; and (3) survival outcomes including OS, CSS, DFS, and RFS. For survival outcomes, hazard ratio (HR) and 95% confidence interval (CI) were directly or indirectly extracted from included studies as previously described [23]. Especially, if HR and 95% CI were both provided by univariate analysis and multivariate analysis, the latter one with higher accuracy would be used. We used the Newcastle-Ottawa scale (NOS), which consisted of 9 scores, to evaluate the quality assessment [24]. Studies with NOS score of >5 were considered to have a high-quality and low risk of bias.

### 2.4 Database validation

The Gene Expression Profiling Interactive Analysis (GEPIA) (http://gepia.cancer-pku.cn/index.html), based on the data from The Cancer Genome Atlas (TCGA), was used to explore the association of NNMT expression with OS or DFS of patients with various cancers or gastrointestinal cancer [25].

### 2.5 Statistical analysis

The Stata 12.0 (StataCorp, College Station, TX, USA) and Review Manager 5.3 (Cochrane Collaboration, London, UK) were used to perform the statistical analysis. Heterogeneity was determined by the Chi square-based *Q* test and *I* ^2^ statistics. Heterogeneity across studies was considered significant when *P* for heterogeneity is <0.05 or *I* ^2^ > 50, as a result, a random-effect model was used. Otherwise, a fixed-effect model was applied. Forest plot based on the *Z* test was generated to determine the association of NNMT expression with OS, DFS, and CP. HR and 95% CI were integrated to show the association between NNMT expression and OS/DFS. Odds ratio (OR) and 95% CI were used to show the association of NNMT expression with CP based on the *Z* test. *P* < 0.05 in the *Z* test indicated that there was a significant association of NNMT expression with survival or CP of human cancers. In addition, we also performed the subgroup analysis to comprehensively explore the association between NNMT expression and OS. Sensitivity analysis was also performed to evaluate the stability of the results. Publication bias referred to the fact that “statistically significant” positive results were more likely to be published than “statistically insignificant” negative or invalid results, which were generally assessed by Begg’s test and Egger’s test, and *P* value less than 0.05 for Begg’s test and Egger’s test indicated that there was a significant publication bias included across studies [26].

## 3 Results

### 3.1 Literature search and selection

**Figure 1:**
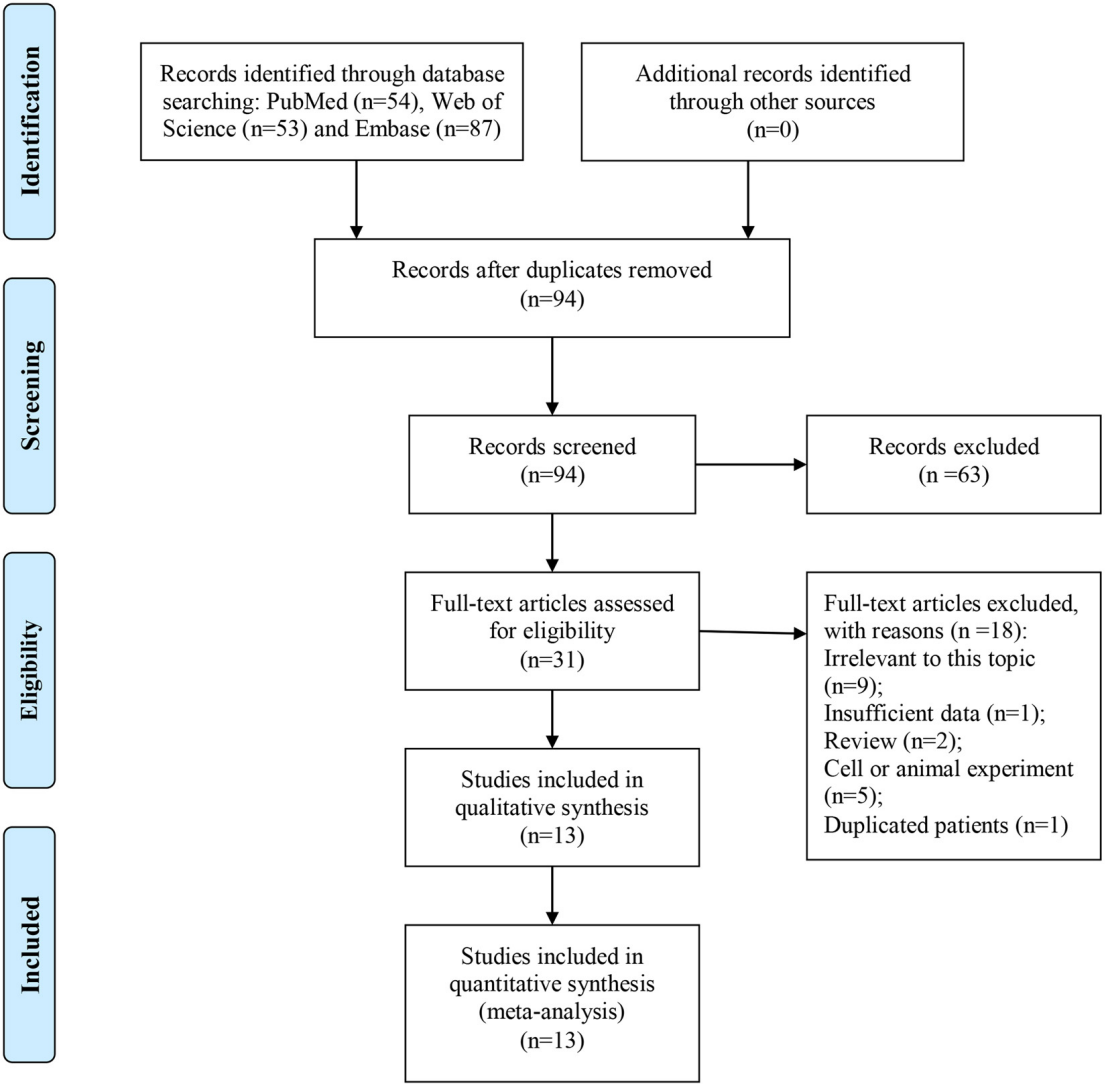
Flowchart of literature search and selection.

The details of literature search and selection are listed in Figure 1. A total of 224 studies were retrieved from common databases, and 13 studies meeting the criteria were finally included in this meta-analysis [9,10,11,12,13,14,15,16,17,18,19,20,21].

### 3.2 Characteristics of included studies

As listed in Table 1, 13 retrospective studies containing 2,591 patients were included in this analysis [9,10,11,12,13,14,15,16,17,18,19,20,21]. Eight studies were conducted in China, two studies were conducted in Japan, one study was conducted in Korea, one study was conducted in America, and one study was conducted in Turkey. The sample size ranged from 22 to 967 among studies. There were 1,455 patients in high NNMT expression group and 1,126 patients in low NNMT expression group. The detection methods included immunohistochemistry, quantitative real time polymerase chain reaction (qRT-PCR), and enzyme linked immunosorbent assay. The details of the detection methods are listed in Table S1. The expression level of NNMT was detected from cancer tissues in 12 studies and serum in 1 study. The following types of cancer were investigated in the current study: endometrial cancer, pancreatic cancer, gastric cancer, esophageal cancer, hepatocellular carcinoma, oral squamous cell carcinoma, colorectal cancer, non-small cell lung cancer, breast cancer, nasopharyngeal carcinoma, and renal cancer. Regarding prognostic outcomes, OS or CSS was reported in nine studies, DFS or RFS was reported in three studies, and CP was reported in nine studies. The association between NNMT expression and OS was determined using multivariate analysis in five studies and univariate analysis in four studies. The adjusted factors in the multivariate analysis of OS are listed in Table S2. NOS score was larger than five in all included studies, which indicated all studies had a relatively high quality and low risk of bias.

**Table 1:** Demographic and clinical characteristics of 13 included studies

Study	Country	Sample size (*n*)	Gender (male/female) (*n*)	NNMT expression			Source	Cancer type	Outcomes	Analysis model	NOS
				High/low (*n*)	High NNMT expression	Detection method					
Akar et al. [9]	Turkey	44	0/44	14/30	Score >80	IHC	Cancer tissue	Endometrial cancer	CSS	M	7
Bi et al. [10]	America	22	NA	14/8	>Median	qRT-PCR	Cancer tissue	Pancreatic cancer	OS	U	6
Chen et al. [11]	China	617	464/153	341/276	Score >120	IHC	Cancer tissue	Gastric cancer	CP and OS	M	8
Cui et al. [12]	China	30	17/13	22/8	Score >3	IHC	Cancer tissue	Esophageal carcinoma	CP	NA	6
Kim et al. [13]	Korea	120	92/28	48/72	Copy number ratio ≥4.40	qRT-PCR	Cancer tissue	Hepatocellular carcinoma	CP, OS, and DFS	U	8
Liao et al. [14]	China	38	37/1	19/19	2+, 3+, 4+	IHC	Cancer tissue	Oral squamous cell carcinoma	CP	NA	6
Song et al. [15]	China	967	573/394	641/326	Score >106	IHC	Cancer tissue	Colorectal cancer	CP, CSS, and DFS	M	9
Ujiie et al. [16]	Japan	109	67/42	27/82	>710 pg/mL	ELISA	Serum	Non-small cell lung cancer	OS	U	7
Wang et al. [17]	China	165	0/165	89/76	Score ≥120	IHC	Cancer tissue	Breast cancer	CP	NA	6
Win et al. [18]	China	124	95/29	62/62	Score >62	IHC	Cancer tissue	Nasopharyngeal carcinoma	CP, CSS, and RFS	M	8
Xu et al. [19]	China	178	104/74	99/79	Score >110	IHC	Cancer tissue	Pancreatic cancer	CP and OS	M	9
Yao et al. [20]	Japan	103	75/28	35/68	>Mean	qRT-PCR	Cancer tissue	Renal cancer	CSS	U	7
Zhang et al. [21]	China	74	48/26	54/20	1+, 2+, 3+	IHC	Cancer tissue	Renal cancer	CP	NA	6

NNMT, nicotinamide *N*-methyltransferase; NOS, Newcastle-Ottawa scale; IHC, immunohistochemistry analysis; qRT-PCR, quantitative real time polymerase chain reaction; ELISA, enzyme linked immunosorbent assay; CSS, cancer-specific survival; OS, overall survival; DFS, disease-free survival; RFS, recurrence-free survival; CP, clinicopathological parameters; M, multivariate; U, univariate; NA, not available.

### 3.3 Association between NNMT expression and OS

**Figure 2:**
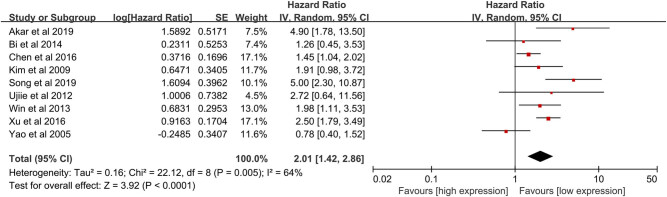
Meta-analysis for the association between NNMT expression and OS indicating that high NNMT expression was significantly associated with worse OS compared to low NNMT expression in cancer patients.

**Figure 3:**
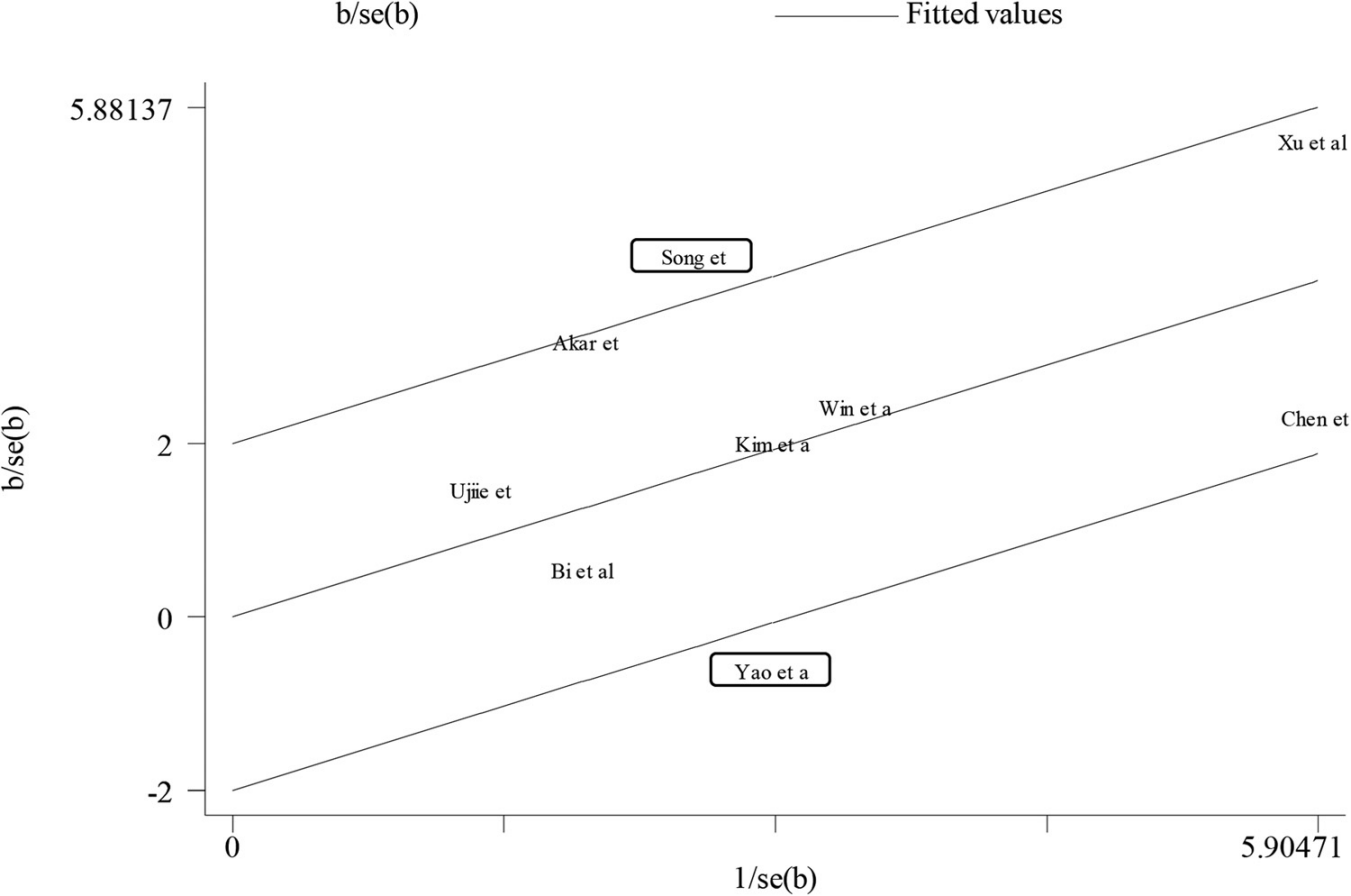
Galbraith plot for the association between NNMT expression and OS showing that studies of Song et al. and Yao et al. were the main sources of heterogeneity based on the Galbraith plot.

**Figure 4:**
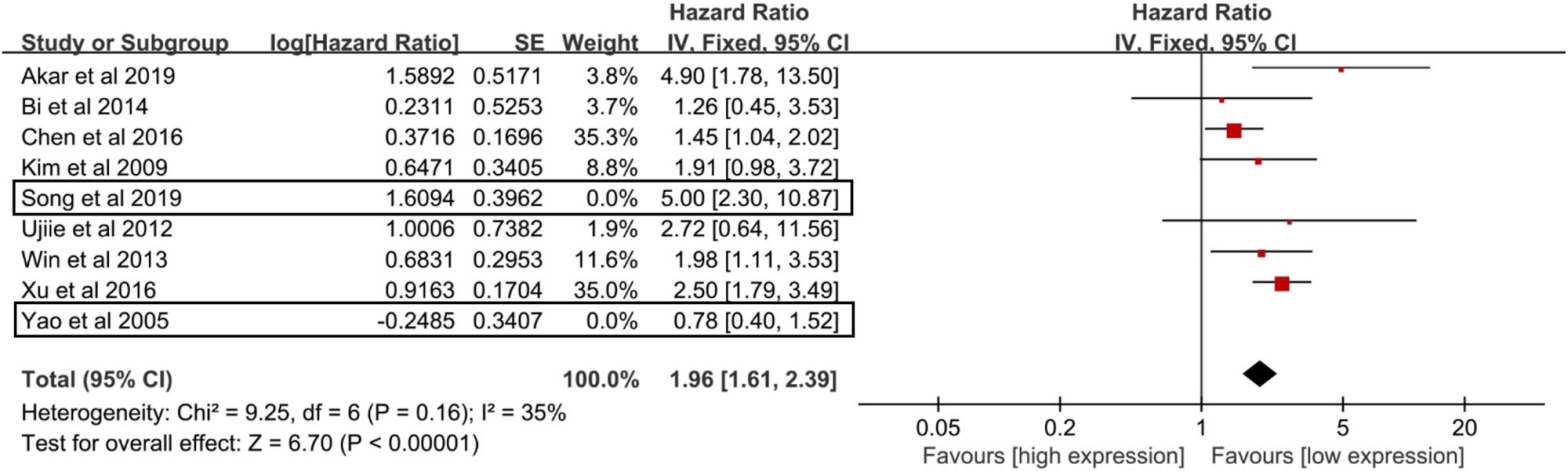
Meta-analysis for the association between NNMT expression and OS after the removal of the studies of Song et al. and Yao et al. also indicating that high NNMT expression was significantly associated with worse OS compared to low NNMT expression in cancer patients.

Nine studies reporting the OS or CSS were included in the pooled analysis [9,10,11,13,15,16,18,19,20]. A random-effect model was used for obvious heterogeneity across studies (*I* ^2^ = 64% and *P* < 0.01), and high NNMT expression was significantly associated with shorter OS compared to low NNMT expression in human cancers (HR = 2.01, 95% CI = 1.42–2.86, and *P* < 0.01) (Figure 2). Studies by Song et al. [15] and Yao et al. [20] were the main source of heterogeneity based on the Galbraith plot (Figure 3). Heterogeneity reduced a lot after the removal of the studies by Song et al. [15] and Yao et al. [20] (*I* ^2^ = 35% and *P* = 0.16), and significant relation between NNMT expression and OS of cancers remained (HR = 1.96, 95% CI = 1.61–2.39, and *P* < 0.01) (Figure 4). We also conduced the subgroup analysis to further determine the association between NNMT expression and OS in cancers. The association between NNMT expression and OS remained significant in most stratified factors (*P* < 0.05), especially gastrointestinal cancers (*P* < 0.01) (Table 2).

**Table 2:** Subgroup analysis of the association between NNMT expression and OS of cancer patients

Factors	Studies (*n*)	HR (95% CI)	*P*	*I* ^2^ (%)	*P* for heterogeneity	Model
Continent						
Asian	7	1.94 (1.33, 2.82)	<0.01*	67	<0.01	Random
Others	2	2.49 (0.66, 9.43)	0.18	71	0.07	Random
Sample size (*n*)						
<150	6	1.78 (1.09, 2.90)	0.02*	53	0.06	Random
≥150	3	2.41 (1.35, 4.27)	<0.01*	81	<0.01	Random
Detection method						
IHC	5	2.48 (1.62, 3.80)	<0.01*	70	0.01	Random
qRT-PCR	3	1.23 (0.80, 1.89)	0.35	42	0.18	Fixed
ELISA	1	2.72 (0.64, 11.56)	0.18	NA	NA	Fixed
Cancer type						
Gastrointestinal cancer	5	2.11 (1.40, 3.18)	<0.01*	65	0.02	Random
Others	4	1.95 (0.90, 4.25)	0.09	70	0.02	Random
Analysis model						
Univariate	4	1.31 (0.87, 1.98)	0.20	34	0.21	Fixed
Multivariate	5	2.48 (1.62, 3.80)	<0.01*	70	0.01	Random
Survival type						
OS	5	1.88 (1.52, 2 .33)	<0.01*	33	0.20	Fixed
CSS	4	2.37 (1.02, 5.55)	0.04*	81	<0.01	Random

NNMT, nicotinamide *N*-methyltransferase; HR, hazard ratio; CI, confidence interval; OS, overall survival; CSS, cancer-specific survival; IHC, immunohistochemistry analysis; qRT-PCR, quantitative real time polymerase chain reaction; ELISA, enzyme linked immunosorbent assay; NA, not available; * *P* value less than 0.05 indicate the significant association between NNMT expression and OS.

### 3.4 Association between NNMT expression and DFS

**Figure 5:**
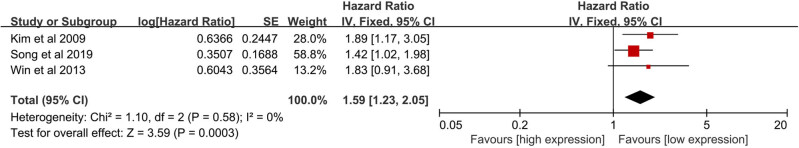
Meta-analysis for the association between NNMT expression and DFS indicated that high NNMT expression was significantly associated with worse DFS compared to low NNMT expression in cancer patients.

Three studies reporting DFS or RFS were pooled to determine the association between NNMT expression and DFS in cancers [13,15,18]. A fixed-effect model was used for no heterogeneity across studies (*I* ^2^ = 0% and *P* = 0.58). Cancer patients with high NNMT expression tended to have a shorter DFS compared to those with low NNMT expression (HR = 1.59, 95% CI = 1.23–2.05, and *P* < 0.01) (Figure 5).

### 3.5 Association between NNMT expression and CP

As listed in Table 3, cancer patients with high NNMT expression tended to have worse tumor differentiation (OR = 1.31, 95% CI = 1.02–1.68, and *P* = 0.03), earlier lymph node metastasis (OR = 1.93, 95% CI = 1.15–3.23, and *P* = 0.01), earlier distant metastasis (OR = 3.18, 95% CI = 1.22–8.26, and *P* = 0.02), and more advanced clinical stage (OR = 1.66, 95% CI = 1.36–2.03, and *P* < 0.01) compared to those with low NNMT expressions. Otherwise, there was no obvious association of NNMT expression with age (*P* = 0.85), gender (*P* = 0.71), tumor size (*P* = 0.14), or T stage (*P* = 0.29).

**Table 3:** Meta-analysis for the association between NNMT expression and CP of cancer patients

Items	Studies (*n*)	Patients (*n*)	OR (95% CI)	*P*	*I* ^2^ (%)	*P* for heterogeneity	Model	Begg’s test	Egger’s test
Age (old/young)	7	1,308	1.05 (0.65, 1.67)	0.85	67	<0.01	Random	1.00	0.81
Gender (male/female)	7	2,110	0.96 (0.80, 1.17)	0.71	15	0.32	Fixed	0.37	0.29
Tumor size (large/small)	2	298	1.99 (0.79, 5.02)	0.14	72	0.06	Random	NA	NA
Differentiation (poor/moderate or well)	7	1,186	1.31 (1.02, 1.68)	0.03*	0	0.49	Fixed	0.13	0.17
T stage (T3 + T4/T1 + T2)	5	1,158	1.44 (0.73, 2.82)	0.29	74	<0.01	Random	1.00	0.47
Lymph node metastasis (yes/no)	7	2,155	1.93 (1.15, 3.23)	0.01*	76	<0.01	Random	0.37	0.26
Distant metastasis (yes/no)	3	960	3.18 (1.22, 8.26)	0.02*	51	0.13	Random	1.00	0.70
Clinical stage (III + IV/I + II)	5	1,864	1.66 (1.36, 2.03)	<0.01*	17	0.30	Fixed	0.81	0.62

NNMT, nicotinamide *N*-methyltransferase; CP, clinicopathological parameters; OR, odds ratio; CI, confidence interval; NA, not available; *P value less than 0.05 indicate the significant association between NNMT expression and CP.

### 3.6 Sensitivity analysis

**Figure 6:**
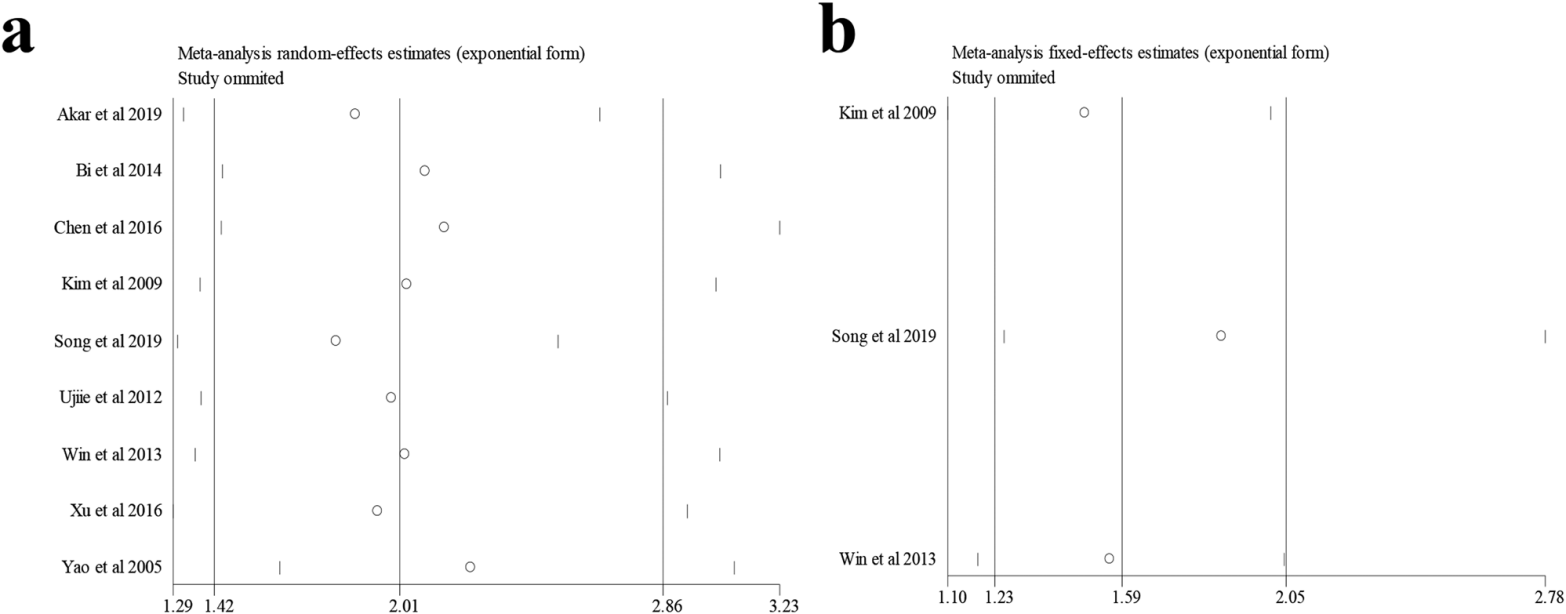
Sensitivity analysis indicated that the meta-analysis results for OS and DFS were reliable: (a) OS and (b) DFS.

Sensitivity analysis was performed by removing any included study in the meta-analysis using Stata 12.0. As shown in Figure 6a, the significant association between NNMT expression and OS was not altered after the removal of any included study, which meant that none of the included studies had the decisive effect on the results. Similarly, the relationship between NNMT expression and DFS remained significant by excluding any included study, which indicated the association between NNMT expression and DFS was reliable (Figure 6a).

### 3.7 Publication bias

**Figure 7:**
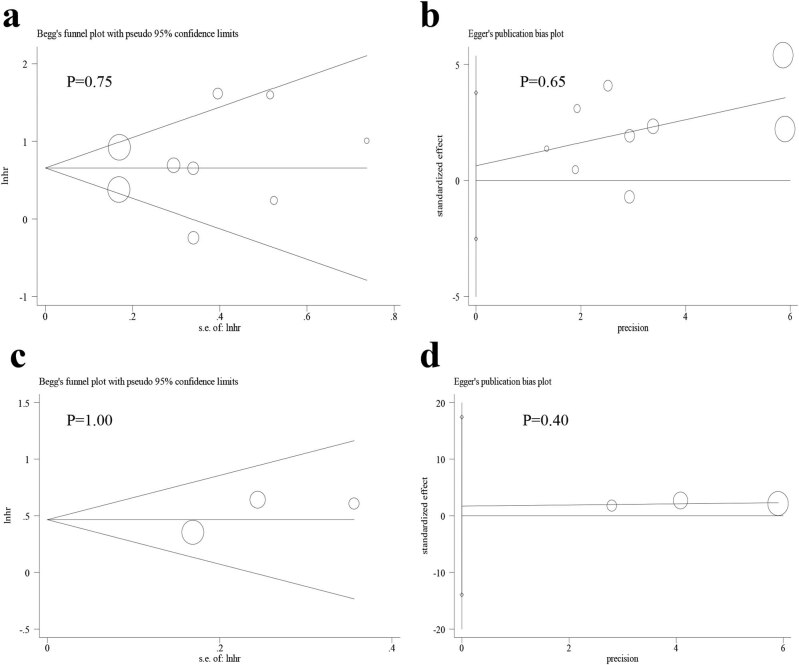
Assessment of publication bias for OS and DFS indicated that there was no obvious publication bias across included studies: (a) Begg’s test for OS; (b) Egger’s test for OS; (c) Begg’s test for DFS; and (d) Egger’s test for DFS.

There was no obvious publication bias across studies in terms of OS (Figure 7a, Begg’s test, *P* = 0.75 and Figure 7b, Egger’s test, *P* = 0.65) and DFS (Figure 7c, Begg’s test, *P* = 1.00 and Figure 7d, Egger’s test, *P* = 0.40). Moreover, no obvious publication bias was observed in the analysis of CP (Begg’s test, *P* > 0.05 and Egger’s test, *P* > 0.05) (Table 3).

### 3.8 Database validation

**Figure 8:**
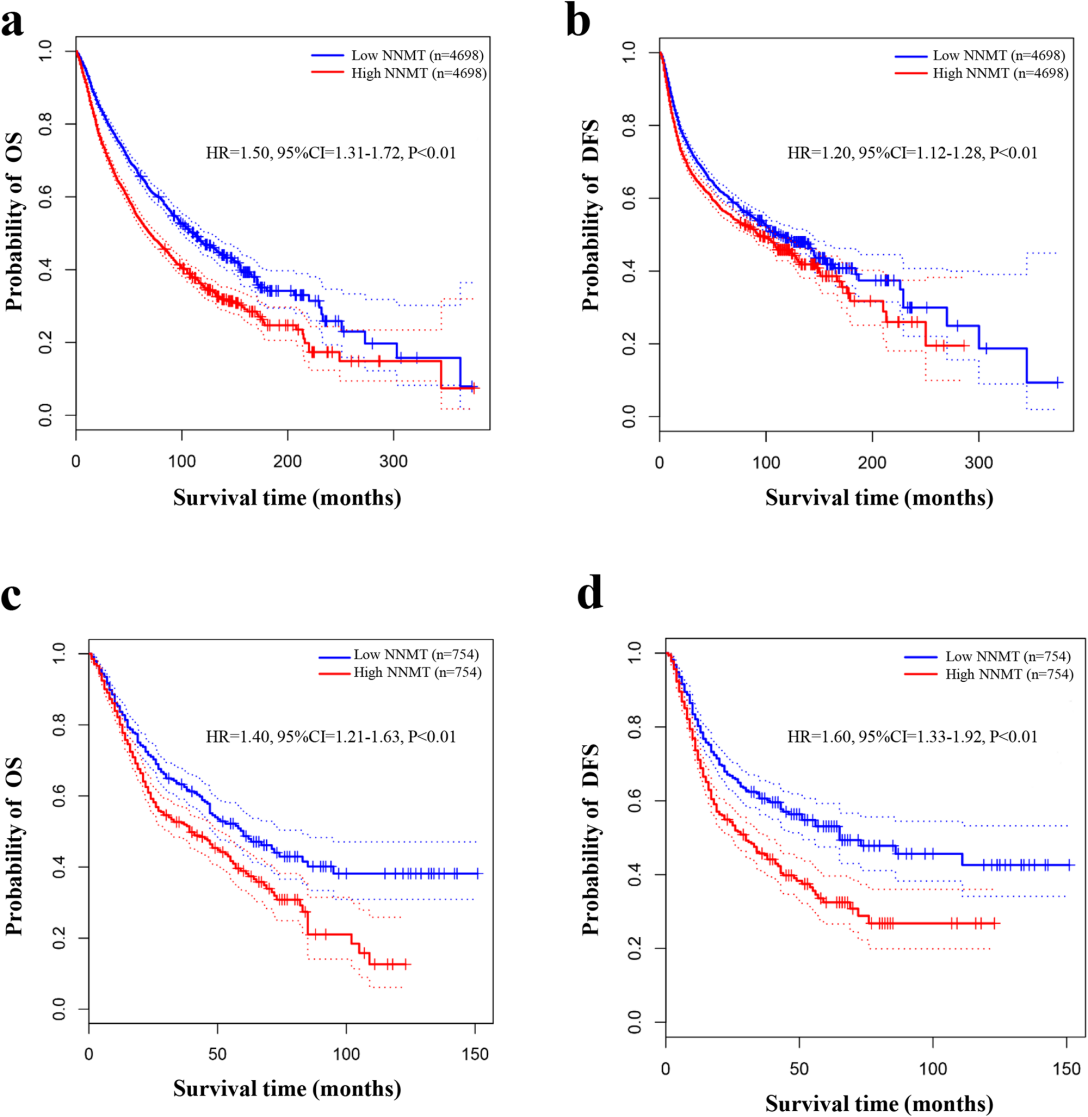
Database validation using TCGA data confirmed that high NNMT expression was significantly associated with worse OS and DFS in various cancers and gastrointestinal cancer: (a) OS for various cancers; (b) DFS for various cancers; (c) OS for gastrointestinal cancer; and (d) DFS for gastrointestinal cancer.

The GEPAI based on the data from TCGA showed that high NNMT expression was significantly associated with shorter OS (HR = 1.50, 95% CI = 1.31–1.72, and *P* < 0.01) (Figure 8a) and DFS compared to low NNMT expression in human cancers (HR = 1.20, 95% CI = 1.12–1.28, and *P* < 0.01) (Figure 8b). Specially, the significant relationship between NNMT expression and prognosis in gastrointestinal cancers was also validated in terms of OS (HR = 1.40, 95% CI = 1.21–1.63, and *P* < 0.01) (Figure 8c) and DFS (HR = 1.60, 95% CI = 1.33–1.92, and *P* < 0.01) (Figure 8d).

## 4 Discussion

Although recent studies have showed that NNMT expression played an important role in the tumor progression and might induce an unfavorable survival outcome for cancer patients, definite conclusion has not been obtained for small sample size and contradictory results of existing evidence [9,10,11,12,13,14,15,16,17,18,19,20,21]. In current study, we performed this meta-analysis to explore the prognostic value of NNMT expression in human cancers, and our study showed that, compared to low NNMT expression, high NNMT expression was significantly associated with shortened OS, DFS, and worse CP, including worse tumor differentiation, earlier lymph node metastasis, earlier distant metastasis, and more advanced clinical stage. Our results showed that there was no obvious publication bias among included studies, which indicated that our results were convincing. Moreover, we also used the TCGA data to validate our findings, and results also showed that high NNMT expression was an unfavorable factor of OS and DFS in human cancers, especially gastrointestinal cancers. Therefore, our study showed that high NNMT expression might predict the worse prognosis of cancer patients, and NNMT expression had the potential capacity to serve as a prognostic factor in various cancers, especially gastrointestinal cancer. Besides, we have noticed that Li et al. performed a similar meta-analysis focusing on the prognostic role of NNMT expression in human cancers [27]. However, some differences between the current study and the study by Li et al. should be mentioned. First, most included data were extracted from glioblastoma patients in the study by Li et al., but we paid more attention to the prognostic value of NNMT expression in the gastrointestinal cancers. Second, we not only explored the association of NNMT expression with cancer survival, but also several vital clinical features (e.g., clinical stage, lymph node metastasis, etc.), which was not performed in the study by Li et al. Third, we used the GEPIA database with a large population to validate our findings, which was not performed in the study by Li et al. Hence, we believe that our study could provide more comprehensive and reliable evidence on this topic.

There are many publications focusing on the underlying mechanism of the prognostic role of NNMT expression in cancers. Wang et al. indicated that NNMT expression could enhance the chemoresistance by sirtuin 1 protein stabilization in breast cancer [17]. Similarly, You et al. also showed that NNMT could promote the tumor progression by stabilizing sirtuin 1 in prostate cancer [28], and Yu et al. indicated that NNMT could inhibit the autophagy induced by oxidative stress through suppressing the AMPK pathway in breast cancer cells [29]. Cui et al. supported the idea that downregulation of NNMT expression could inhibit the migration and epithelial-mesenchymal transition of esophageal cancer trough the Wnt/β-catenin pathway [12]. With respect to gastric cancer, Liang et al. suggested that NNMT could promote the epithelial-mesenchymal transition of cancer cells by activating transforming growth factor-β1 expression [30]. Besides, Bi et al. found that miR-1291-altered PANC-1 cell function could increase the expression level of *N*-methylnicotinamide level and NNMT expression, and they drew the conclusion that NNMT might be indicative of the extent of pancreatic cancer [10]. A study conducted by Palanichamy et al. showed that NNMT could inhibit the tumor suppressor enzyme PP2A by reorganizing the methylome and concomitantly activate the serine/threonine kinases [31]. Moreover, Zhang et al. found that downregulation of NNMT could induce the apoptosis via the mitochondria-mediated pathway in breast cancer cells [32]. The research of Xie et al. manifested that NNMT could enhance the resistance to 5-fluorouracil through inhibition of the ASK1-p38 MAPK pathway in colorectal cancer cells, and further result in the poor prognosis [33].

Our findings, based on the existing evidence, showed that high NNMT expression was associated with worse prognosis of cancer patients, and our findings were further confirmed by the TCGA data. Therefore, the conclusion of our study was reliable and could provide the valuable evidence on the clinical decision-making. Nevertheless, our study was not without limitations. First, all included studies had a retrospective design, which induced the selection bias of patients and further affected our results. Second, prognostic value of NNMT expression was evaluated in all types of cancer because of limited sample size of one specific cancer, which might limit the promotion of our findings. Third, heterogeneity was obvious in some analyses (e.g., clinical stage and lymph node metastasis), and a random-effect model has to be used, which might reduce the accuracy of our results. Fourth, Cox multivariate analysis to determine the independent prognostic factors for the prognosis of cancers failed to be performed in this meta-analysis, because all data in this meta-analysis were extracted from published studies and the data of individuals were unavailable for us. To eliminate these limitations, future studies that are well-designed with enough follow-up period should be performed to determine the prognostic role of NNMT expression in cancers.

## 5 Conclusion

High NNMT expression was significantly associated with shorter OS, DFS, and worse clinicopathological features compared to low NNMT expression in various cancers. Therefore, NNMT expression could serve as a promising prognostic factor and potential therapeutic target in various cancers, especially gastrointestinal cancer.

## Abbreviations

CI: confidence interval
CP: clinicopathological parameters
CSS: cancer-specific survival
DFS: disease-free survival
GEPIA: gene expression profiling interactive analysis
HR: hazard ratio
NNMT: nicotinamide *N*-methyltransferase
NOS: Newcastle-Ottawa scale
OR: odds ratio
OS: overall survival
RFS: recurrence-free survival
TCGA: The Cancer Genome Atlas
